# Editorial: Advances in comprehensive treatment of postmenopausal osteoporotic fractures

**DOI:** 10.3389/fmed.2024.1378312

**Published:** 2024-02-13

**Authors:** Yori Endo, Luisa Weber, Jessica Mroueh

**Affiliations:** ^1^Department of Surgery, Brigham and Women's Hospital, Harvard Medical School, Boston, MA, United States; ^2^Division of Hand, Plastic and Aesthetic Surgery, Ludwig-Maximilians-Universität München, München, Germany

**Keywords:** osteoporosis, osteoporotic fracture, vertebral fractures in osteoporosis, management of fractures, hip fractures

Postmenopausal osteoporotic fractures (PMOF) can affect up to 50% of postmenopausal women (1). The decrease in estrogen levels and the change in the local bone microenvironment leads to enhanced osteoclastic activity and osteoporosis. The bone loss in the first 5–7 years following menopause increases at an annual rate of 1–5%, resulting in a reduction of trabecular bone density and an increasing tendency for fractures, most significant of which are thoracolumbar osteoporotic compression fractures, hip fracture and Colles' fracture. The current treatment methods for PMOF are limited mostly to internal fixation combined with anti-osteoporotic drug treatment. However, due to the increasing loosening of the bone microenvironment in patients, the rate bone non-union is as high as of 13.5–19.6% (2, 3). Therefore, there is a critical need for developing more effective therapies for PMOF. This Research Topic covers basic and clinical research on PMOF focusing on advances in treatment methods, understanding of molecular mechanisms, and cross-disciplinary applications.

Vertebral compression fractures become increasingly common after menopause as osteoporosis of vertebrae progresses. The current treatment options have been limited mostly to conservative management with pain medication, back brace and bed rest. In recent years, minimally invasive implants are becoming one of the viable therapeutic options. Technological advancement has improved the available implants and their safety, and they may be preferred for the treatment of those with advanced osteoporosis in order to prevent further compression fractures (4). The treatment with implants needs considerations given to the risk of neurological damage, implant biocompatibility, and compatibilities of the mechanical properties between the implant and vertebral body. Therefore, designing new implants should ensure appropriate mechanical strength to give vertebral stability and adequate biocompatibility. Luo et al. summarized and compared the available implants in this Research Topic, and concluded that Jack, SBE and Spine Jack, VBS and Osseofix are able to maintain or restore vertebral height, while Kiva and StaXx FX can strut the compressed vertebra with more precision. They also concluded that Vesselplasty and Optimesh have lower leakage incidences, and recommend that the V-strut system is used for osteolytic fractures caused by vertebral tumors. They recommend that advantages and disadvantages of these implants are individually considered and weighed against each other for each patient's case for an optimal delivery of personalized care.

Surgical interventions for hip fractures such as joint replacements, where bones are sectioned to insert a prosthesis, put patients at risk of postoperative bleeding complications. Bones have a network of blood vessels within their trabecular structure that can rapture to cause osseus hemorrhage, which is difficult to control because of the deep location of the bleeding source within the tissue. The perioperative hemorrhage control during orthopedic surgeries is therefore important and must be effective. In this Research Topic, Li et al. examined the efficacy of bone wax, a combination of beeswax and softening agents, to seal the bleeding site in total hip arthroplasties in a randomized controlled trial with 104 patients. The application of the aforementioned improved total blood loss, benefitting patients with stable hemoglobin levels and a shorter postoperative hospital stay. Compared to the other sealing techniques, they report that the bone wax did not demonstrate unfavorable characteristics such as rapid degradation rates, harmful effects or delays in wound healing, highlighting its suitability for potential use in clinic.

By increasing the susceptibility to bone fractures, osteoporosis poses a significant health concern especially for postmenopausal women. Up to 30% of patients experience subsequent fractures within the first 2 years after an initial fracture, highlighting the importance of implementing an effective prophylactic strategy for fracture prevention (5, 6). Previously, the Fracture Risk Assessment Tool (FRAX) and the QFractureScore were introduced to evaluate the risk of initial fracture for patients with osteoporosis. However, the assessment for the risk of subsequent contralateral hip fractures lacked an appropriate tool, despite these fractures being life-threatening for the elderly. Liang et al. developed a predictive nomogram examining the most significant eight predictors based on data from a single-center retrospective study of 734 elderly hip-fracture patients. This multivariate model predicts the risk of subsequent contralateral fracture based on co-morbidities demographics, and laboratory characteristics. They argue that the model enables clinicians to tailor perioperative management and implement early preventive measures for each patient following within 2 years of the operation. Their method showed a high level of consistency and discrimination, demonstrating its potential as a useful clinical tool to be incorporated into prophylactic therapies for contralateral hip fractures.

The Hoffa fracture is a rare type of coronal fracture of the femoral condyle, accounting only for 0.1% of all fracture types (7). It is understood be caused by vertical forces exerted from the femoral condyle on the posterior aspect of the tibial plateau when the knee is in a flexed position at the time of injury. Due to the specificity and scarcity of this type of fracture, the optimal treatment is yet to be established. Xue et al. developed a simplified 3D model of the fracture that allows for simulation of the facture and various methods of internal fixation using plates and screws under physiological load, and compared four different fixation methods using computational models (8–10). Their data indicate that lateral plate combined with posterior plate fixation is the most effective for Hoffa-like tibial plateau fractures of the four methods simulated, offering an insight into optimized fixation method.

## Author contributions

YE: Writing – original draft, Writing – review & editing. LW: Writing – original draft. JM: Writing – original draft.
